# Management of soil pH promotes nitrous oxide reduction and thus mitigates soil emissions of this greenhouse gas

**DOI:** 10.1038/s41598-019-56694-3

**Published:** 2019-12-27

**Authors:** Catherine Hénault, Hocine Bourennane, Adeline Ayzac, Céline Ratié, Nicolas P. A. Saby, Jean-Pierre Cohan, Thomas Eglin, Cécile Le Gall

**Affiliations:** 1 Agroécologie, AgroSup Dijon, INRAE, Univ. Bourgogne Franche-Comté, F-21000 Dijon, France; 2 URSOLS, INRAE, 45075 Orléans, France; 3 Infosol, US1106, INRAE, 45075 Orléans, France; 4 ARVALIS- Institut du Végétal Route de Châteaufort – RD 36 – ZA des Graviers, 91190 Villiers le Bacle, France; 5 ADEME, Direction Bioéconomie et Energies Renouvelables, Service Forêts, Alimentation et Bioéconomie, F-49000 Angers, France; 6 TERRES INOVIA, Avenue Lucien Brétignières, 78850 Thiverval Grignon, France

**Keywords:** Element cycles, Environmental impact

## Abstract

While concerns about human-induced effects on the Earth’s climate have mainly concentrated on carbon dioxide (CO_2_) and methane (CH_4_), reducing anthropogenic nitrous oxide (N_2_O) flux, mainly of agricultural origin, also represents an opportunity for substantial mitigation. To develop a solution that induces neither the transfer of nitrogen pollution nor decreases agricultural production, we specifically investigated the last step of the denitrification pathway, the N_2_O reduction path, in soils. We first observed that this path is mainly driven by soil pH and is progressively inhibited when pH is lower than 6.8. During field experiments, we observed that liming acidic soils to neutrality made N_2_O reduction more efficient and decreased soil N_2_O emissions. As we estimated acidic fertilized soils to represent 37% [27–50%] of French soils, we calculated that liming could potentially decrease France’s total N_2_O emissions by 15.7% [8.3–21.2%]. Nevertheless, due to the different possible other impacts of liming, we currently recommend that the deployment of this solution to mitigate N_2_O emission should be based on local studies that take into account agronomic, environmental and economic aspects.

## Introduction

Agriculture is an important anthropogenic source of nitrous oxide (N_2_O), which has a global warming potential approximately 300 times higher than that of carbon dioxide (CO_2_) on a molar basis and a 100-year period^1^. In 2011, N_2_O accounted for 6% of radiative forcing change since 1750^2^. Arable soils emit more N_2_O to the atmosphere than any other anthropogenic source^3^, i.e., 4.2 teragrams (Tg) of the global anthropogenic flux of 8.1 Tg N_2_O-N yr^−1^. Reducing soil N_2_O flux represents a substantial mitigation opportunity for agriculture to reduce its contribution to greenhouse gas emissions, and it also represents an opportunity to reduce radiative forcing at the global scale. The diversity of microbial metabolic pathways in soils provides a wealth of processes that form or consume N_2_O^4^. The microbial processes of denitrification in anoxic conditions and nitrification in the presence of oxygen (O_2_) are generally admitted to be the main sources of N_2_O in managed and natural soils^4^. The last step of the denitrification process (occurring classically or associated with co-denitrification or nitrification-denitrification)^4^, i.e. the reduction of N_2_O to N_2_, is currently the only known pathway for the terrestrial removal of N_2_O. Recently, in France, the potential of ten measures for mitigating agricultural greenhouse gas emissions was evaluated^5^ using a methodology with the calculation of both the specific potential abatement (i.e. the difference in emissions between a reference situation – baseline – and a situation where a specific measure is applied, related to the emissions of the reference situation) and the potential applicability (i.e. the surface area on which the application of the measure is technically possible^5^) for each measure. For example, one of these measures was to reduce the use of synthetic mineral fertilizers leading to an annual mitigation potential of approximately 0.013 Tg N-N_2_O (6 Tg CO_2_e – equivalent CO_2_ using the 100-year global warming potential value published in 2006, i.e. 298) in 2030 at the scale of mainland France. Although ref. ^3^ also considered that N_2_O abatement could preferentially be achieved by attenuating known sources of N_2_O emissions since N_2_O has no significant terrestrial sink, we hypothesized in this study that strategies to mitigate N_2_O emissions from agricultural soils could be based on the promotion of the last step of the denitrification pathway, i.e. the reduction of N_2_O into N_2_. There are different advantages of managing soil N_2_O reduction for mitigating N_2_O emissions: (i) the N_2_ final form of this transformation is inert, and no transfer of nitrogen pollution occurs during N_2_O reduction; (ii) certain studies on this specific step of the nitrogen cycle, catalysed by the N_2_O reductase enzyme encoded by the *nosZ* gene, have provided conclusive results regarding the quantitative detection of the abundance of the *nosZ* gene and regarding the proportion of denitrifiers in relation to total bacteria in soils^6^, and also, an inhibitor of this transformation (acetylene) can be used in the laboratory for experimental purposes^7^; (iii) previous reports have shown variations in the functioning of this transformation from soil to soil and suggest a link between a soil’s capacity to reduce N_2_O and the intensity of *in situ* N_2_O emissions^8^; and (iv) there is no interference between a soil’s capacity to reduce N_2_O and agricultural production. Moreover, the capacity of soil to reduce N_2_O can be characterized through a laboratory test (ISO/TS 20131–2) derived from the laboratory protocol used in ref. ^8^ that provides quantitative indicators (r_max_ and index) with rules of interpretation (the higher the r_max_ and index are, the lower the capacity of soil to reduce N_2_O). We therefore developed this study to test the following hypotheses: (i) the capacity of soil to reduce N_2_O affects the intensity of *in situ* soil N_2_O emissions^8^; (ii) the capacity of soil to reduce N_2_O is determined by biogeochemical properties, i.e., the *a priori* soil pH and soil organic carbon content^9^; and (iii) the management of these properties can modify the capacity of a given soil to reduce N_2_O into N_2_ and therefore the intensity of soil N_2_O emissions at the field scale. During this study, we successively (i) examined in the laboratory the extent to which the capacity of soil to reduce N_2_O is determined by soil biogeochemical properties, (ii) tested under field conditions how the management of soil pH and soil organic matter affects the capacity of soil to reduce N_2_O and the intensity of soil N_2_O emissions, and (iii) calculated at the national scale the N_2_O emission mitigation that could be achieved by managing relevant soil biogeochemical properties.

## Results

### Biogeochemical determinism of the capacity of soil to reduce N_2_O

**Figure 1:**
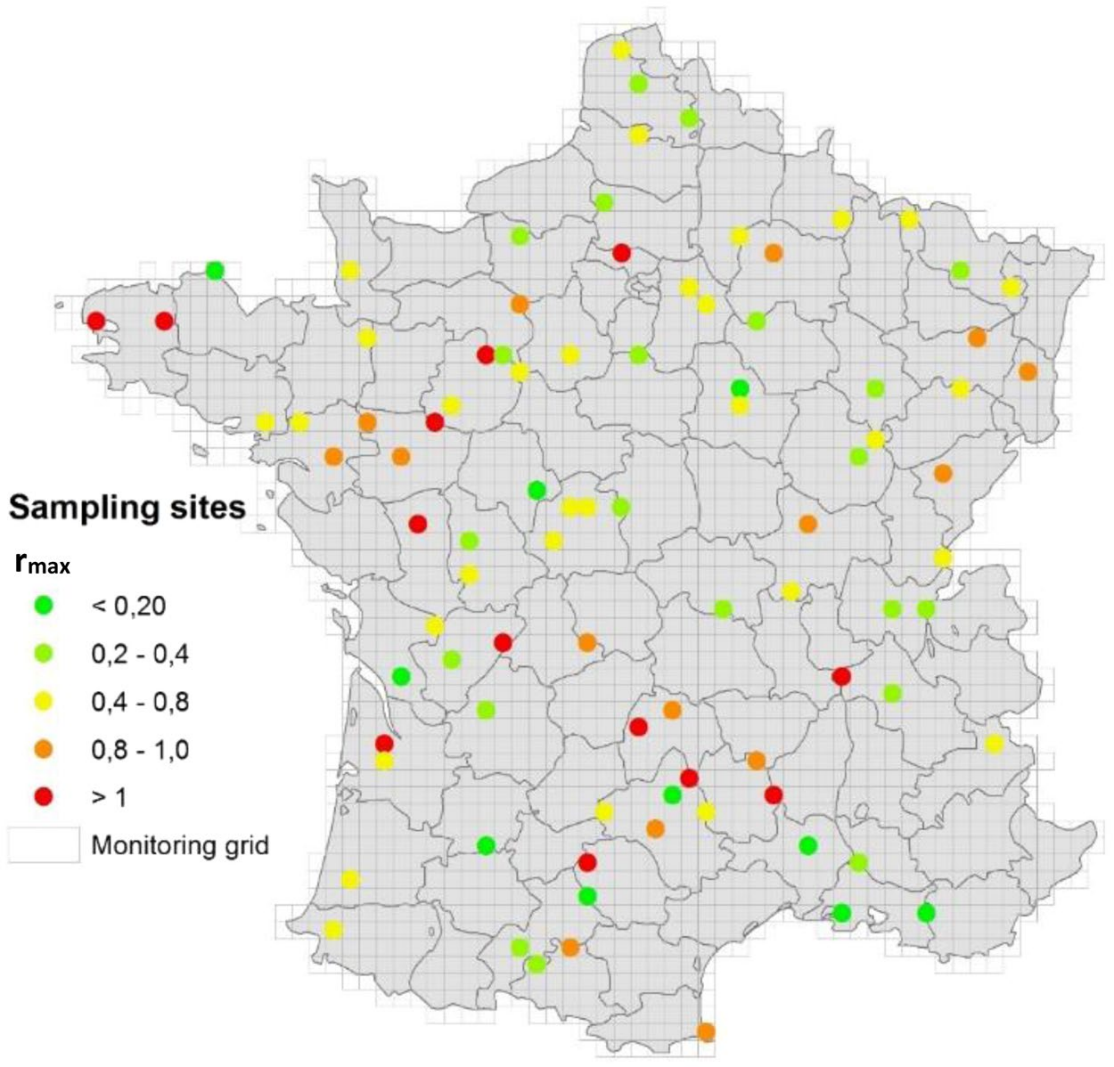
The capacity of soils to reduce N_2_O, expressed as the r_max_ indicator and shown on the RMQS grid.

We sampled 90 selected sites within the framework of the French Soil Monitoring Network (RMQS – Réseau de Mesure de la Qualité des Sols)^10^. Amongst the soil properties routinely analysed during the RMQS program (Supplementary Information 1_1), those used for the selection of sites were pH, soil organic carbon content, land use and geographical position. The distributions of these variables among the selected sites were consistent with the total RMQS (Supplementary Information 1_2). We determined the capacity of these soils to reduce N_2_O through soil laboratory incubations according to ISO/TS 20131-2 derived from ref. ^8^ to determine the indicators r_max_ and index (respectively, the maximum ratio of the accumulated N_2_O during incubation and a combination of r_max_ and the time of accumulation of N_2_O during incubation). During all the soil incubations, we observed the production and consumption of N_2_O, at soil specific rates. We did not observe any variation of N_2_O concentration over time for the treatment with N_2_O and C_2_H_2_, validating that in the experimental conditions imposed, C_2_H_2_ totally inhibits N_2_O reduction. Some examples of the calculation of the r_max_ and index indicators are presented in Supplementary Information 2. All r_max_ and index values obtained are shown on the RMQS grid (Fig. 1).

Taking into account the meaning of these indicators, the rule of interpretation proposed in ISO/TS20131-2 and the concept of soil phenotypes^11^, we *a priori* classified the soils into three phenotype classes: [soil]_PhN2Ored+,_ able to reduce N_2_O (r_max_ and index lower than 0.4 and 30, respectively); [soil]_PhN2Ored−_, unable or nearly unable to reduce N_2_O (r_max_ higher than 0.8 or index higher than 50); and others, called [soil]_PhN2Ored+/−_, that have an intermediate capacity to reduce N_2_O. Among the 90 subset sites, we found that 36 sites (40%) were poorly able to reduce N_2_O ([soil]_PhN2Ored−_), while 29 sites (32%) were very efficient at reducing N_2_O ([soil]_PhN2Ored+_) and 25 sites (28%) were intermediate ([soil]_PhN2Ored+/−_). The detailed results are presented in Supplementary Information 3.

Soil pH appeared to be the factor that explained most of the variability of a soil’s capacity to reduce N_2_O, explaining 61% and 59% of r_max_ and index variability, respectively, for all the statistical methods used (linear regression, partial least square regression^12^, generalized boosted models^13^ and classification and regression trees coupled to factorial discriminant analysis^14,15^). Contrary to one of our hypotheses, the soil organic carbon content did not appear to be a predictor of the capacity of a soil to reduce N_2_O. We observed that a soil’s capacity to reduce N_2_O can be predicted using a partial least square (PLS) model based on soil pH, the cation exchange capacity (CEC) determined by the cobalt-hexamine method described in NF X31-130 (cmol kg^−1^) and ref. ^16–18^ and, the soil clay content (g kg^−1^). The following empirical pedotransfer equations were obtained (Eqs. 1 and 2, with the r value being the linear correlation coefficient between the measured and predicted values of each indicator):

1 $${r}_{max}=-\,0.4\,pH+0.026\,CEC-0.001\,Clay+3.13\,{\rm{r}}=0.88$$

2 $$Index=-\,94.18\,pH+7.47\,CEC-0.25\,Clay+645.12\,{\rm{r}}=0.90$$

**Figure 2:**
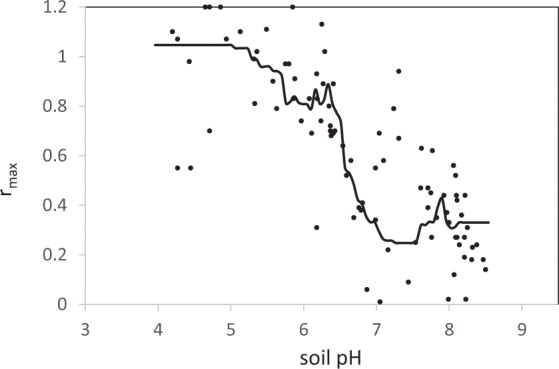
The experimental points of r_max_ against soil pH and the function relating r_max_ and soil pH obtained by the GBM.

Root mean square error is 0.17 and 2.05 for Eqs. 1 and 2, respectively. Moreover, a generalized boosted model (GBM) linked pH and r_max_ through a step function (Fig. 2).

The predictive quality of this function was estimated using a leave-one-out cross-validation step with 300 repetitions. We estimated the determination coefficient to be 0.64 and RMSE 0.22. This function suggests that (i) soils with pH < 6.4 have a very low capacity to reduce N_2_O (r_max_ > 0.8), and (ii) soils with pH > 6.8 are able to reduce N_2_O efficiently (r_max_ < 0.4), and that the path of N_2_O reduction becomes efficient across a pH range of only 6.4 to 6.8.

Classification and regression trees (CART) combined with factorial discriminant analysis (FDA) revealed 8 index-pH classes with a clearly consistent transition where pH was between ~6.4 and ~7 (Supplementary Information 4_1) for soils exhibiting a high index compared to soils exhibiting a low index.

Taken together, the results from this statistical processing converge to reveal (i) that pH can be used as a predictor of a soil’s capacity to reduce N_2_O and (ii) that N_2_O reduction is poor when pH is lower than 6.4 and totally efficient at a pH higher than 6.8–7 with a transient zone of efficiency in the 6.4–6.8 pH range, these limits being consistent with the *a priori* proposed classes of phenotypes.

### Effect of liming on soil *in situ* N_2_O emissions

**Figure 3:**
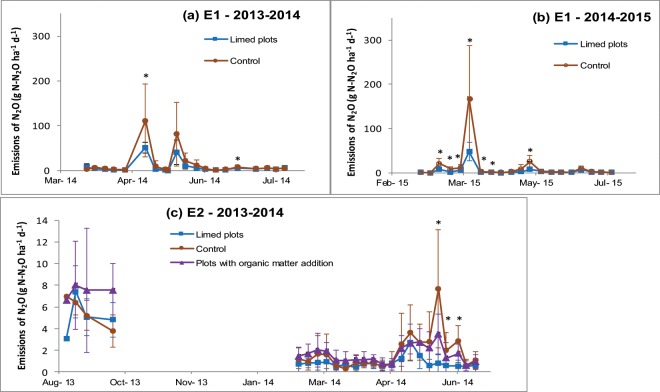
*In situ* N_2_O emissions measured (mean and standard deviations obtained from the 9 replicates per treatment) in E1 (upper panel) and E2 (lower panel). *Indicates significant (p < 0.05) differences for results obtained on limed plots and control ones. In E1, liming events were of 1 t CaO applied in September 2013 and 2014. Nitrogen fertiliser was spread on 14/05/2014 (60 kg of N ha^−1^) and on 09/03/2015 (70 kg of N ha^−1^), on 30/03/2015 (50 kg of N ha^−1^), and on ^1^0/05/2015 (60 kg of N ha^−1^). In E2, liming event was of 1.5 t CaO in September 2013 and organic matter was applied at the same date. Mineral fertilisers were spread on 2/05/2014 (50 Kg N ha^−1^) and on 9/05/2014 (50 Kg N ha^−1^). The “pig manure” plots did not receive the second input.

*In situ* experiments (E1 and E2) were performed to test how the management of soil pH and of soil carbon content affects the capacity of soil to reduce N_2_O and thus the level of soil N_2_O emissions. E1 was a two-year experiment testing the liming effect, and E2 was a one-year experiment testing the individual effects of liming and carbon addition on N_2_O emissions and the capacity of soil to reduce N_2_O. During these experiments, we classically observed peaks of N_2_O emissions after N fertilizer applications (Fig. 3). These peaks reached different levels, due to site and annual climate conditions. The dynamics of N_2_O emissions were observed to be significantly different in limed plots compared to control ones in E1 (2013–2014 and 2014–2015) and in E2. N_2_O emission peaks were clearly lower in limed plots compared to the controls. During E2, the addition of organic C did not affect the overall dynamics of N_2_O emissions.

**Figure 4:**
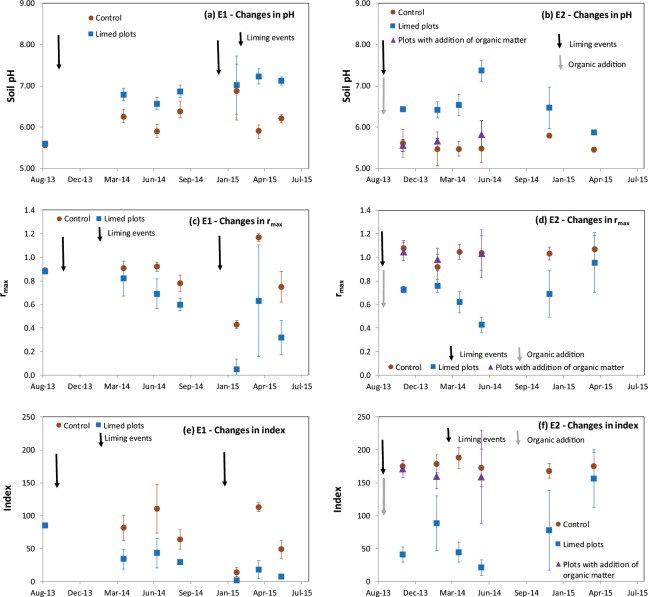
Changes in the indicators (pH, r_max_ and index) of the capacity of soil to reduce N_2_O during E1 (left) and E2 (right). Error bars represent standard deviations (n = 3).

Soil temperature, moisture, and mineral nitrogen contents were not affected by liming at either site. We observed a peak of ammonium content just after the addition of organic matter in E2 while the other parameters (soil temperature, moisture and nitrate contents) were not affected by the treatments throughout the experiment (Supplementary Information 5). Soil bulk density was 1.45 at both sites which nevertheless differed with respect to soil moisture. This was always low in E2 (<18%) while it reached values as high as 25% in E1. This corresponded to a WFPS (water filled pore space)^19^ lower than 60% in E2 and with values as high as 80% in E1, suggesting that the denitrification process would have occurred rarely and inefficiently in E2, though temporarily and intensively in E1. While differences in pH were observed between the control and limed plots in both experiments, we also observed a difference in the capacity of soil to reduce N_2_O (Fig. 4). Both indicators (r_max_ and index) decreased as pH increased, indicating that the capacity of soil to reduce N_2_O becomes more efficient at a higher pH, consistent with the results obtained during the laboratory experiments. On the other hand, the addition of organic C in E2 did not affect the capacity of the soil to reduce N_2_O.

Using Eq. 3, we determined the potential abatement effect of liming on N_2_O emissions. The values obtained were 49% in 2013–2014 and 66% in 2014–2015 in E1 and 26% in 2013–2014 in E2, leading to a rough estimation (median of previous values) of the potential abatement of N_2_O emissions due to liming of 49%.

3 $$abatement=\frac{mean{({N}_{2}O)}_{control}-mean{({N}_{2}O)}_{limed}}{mean{({N}_{2}O)}_{control}}$$

With

*mean(N*_2_*O)*_*control*_: Annual mean of N_2_O emissions measured in the control plots (g N-N_2_O ha^−1^ y^−1^)

*mean(N*_2_*O)*_*limed*_: Annual mean of N_2_O emissions measured in the limed plots (g N-N_2_O ha^−1^ y^−1^)

The results obtained in the field were consistent with the results obtained in the laboratory. On both scales, we observed that increasing pH promoted N_2_O reduction with pH increase, consistent with a decrease of *in situ* N_2_O emission. Also on both scales, we did not detect any significant effect of soil organic carbon on N_2_O reduction or on *in situ* N_2_O emission. Moreover, the effect of increasing soil pH on both the capacity of soil to reduce N_2_O and on soil *in situ* N_2_O emissions occurred almost immediately.

Consistent simulations (differences between the dynamics of measures and simulations were not significant, RMSE was lower than 2) obtained with the NOE algorithm (Supplementary Information 5) suggest that denitrification was the main process involved in N_2_O emission in E1 while nitrification was the main process involved in N_2_O emission in E2. This difference was mainly due to the differences in soil water filled pore space (WFPS) between these two situations, since it was high in the poorly drained situation in E1 and low in the well-drained situation in E2. Introducing the specific measured r-values for describing the N_2_O reduction, made it possible to obtained reasonably well predicted abatement values.

### Assessment of the mitigation potential at the national scale

We then assessed the potential applicability of these results at the national scale, across all of France, assuming that liming can be applied to all fertilized cultivated or grassland soils. Using the total RMQS database composed of 2145 sites, we assessed that fertilized cultivated and grassland soils represent 41% and 19%, respectively, of the soils in France, together accounting for 60% of soils in France and corresponding to weighted contribution of 68% and 32% for cropped and grassland soils, respectively. Different approaches (“CART-FDA”, “cumulative frequency”, “map_rmqs”) were used, each involving a combination of the three indicators (pH, r_max_ and/or index) of the capacity of soil to reduce N_2_O. All three approaches were developed into three steps: (i) the quantification of some of these indicators at the national scale, (ii) the thresholding per phenotype, and (iii) the weighting per land use. Intermediate calculations of these three approaches are presented in Supplementary Information 4.

The application of the eight index-pH classes defined by CART-FDA (Supplementary Information 4_1) to the total RMQS database indicated that 35% of the cropped soils and 65% of the grassland soils had an index higher than 30, leading to an estimation of a potential applicability of 45% of the fertilized soil surface.

The “cumulative frequency” approach (Supplementary Information 4_2) indicated a potential applicability of 61% of the fertilized soil surface, with 46% of soils being [soils]_PhN2Ored−_ and 15% being [soils]_PhN2Ored±_.

The “map_rmqs approach” (Supplementary Information 4_3) indicated that the area of soils with a low capacity to reduce N_2_O ([soil]_PhN2Ored−_) was 38%, while that of soils that very efficiently reduced N_2_O ([soil]_PhN2Ored+_) was 15%, and soils that were intermediate ([soil]_PhN2Ored±_) accounted for 47%.

After estimating the potential applicability based on different approaches (Table 1), we applied the values of potential abatement. We used values observed during previous field experiments. We distinguished full potential abatement (49%) for [soils]_PhN2Ored−_ and half of full potential abatement (25%) for [soils]_PhN2Ored+/−_. We introduced a confidence interval for this estimation, corresponding to the low and high potential abatement values we observed previously, i.e., 26 and 66%.

**Table 1:** Estimated benefit of liming as a method for N_2_O emission mitigation obtained by the different approaches “CART-FDA, “cumulative frequencies” and “map_RMQS”, using the indicators pH, r_max_ and index.

Approaches	Potential abatment	Representativity of fertilised soils	Potential applicability			Potential of mitigation of soil N_2_O emission at the France Scale		Contribution of soil N_2_O emissions to total national emissions of anthropogenic GHG	Potential for mitigating N_2_O emission inside the total national emissions of anthropogenic GHG
			Total	Soil PhN_2_Ored^−^	Soil PhN_2_Ored^+/−^				
		%	%	%	%	%	%	%	%
“CART-FDA”	Median	60		45		49		6.5	0.86
	Lowest	60		45		26		6.5	0.46
	Highest	60		45		66		6.5	1.16
“Cumulative Frequencies”	Median	60	61	46	15	49	25	6.5	1.02
	Lowest	60	61	46	15	26	13	6.5	0.54
	Highest	60	61	46	15	66	33	6.5	1.38
“Map from RMQS”	Median	60	84	38	47	49	25	6.5	1.13
	Lowest	60	84	38	47	26	13	6.5	0.62
	Highest	60	84	38	47	66	33	6.5	1.52
Global estimation (median of values obtained per each approach							15.73	6.50	1.02

The potential for mitigating N_2_O emissions by liming acidic fertilized soils was then assessed as 15.7% (8.3–21.2%) at the national scale in France. As soil N_2_O emissions were estimated to represent 6.5% of the total national emissions of anthropogenic greenhouse gas on a CO_2_e basis, we assessed a mitigation of 1.02% (0.5–1.4%) of this total national emission of anthropogenic greenhouse gas. This is in the same order of magnitude as the assessment of mitigation based on the decreased use of nitrogen fertilizer proposed by ref. ^5^.

## Discussion

Using the framework of a potential “Technical Readiness Level Scale” dedicated to the reduction of GHG emissions, this study allowed for (i) advancing the concept of managing the microbial N_2_O reduction path, based on investigations conducted in the laboratory, (ii) completing the critical stage of field demonstrations and therefore (iii) calculating the potential mitigation at the national scale, consistent with international agreements on climate change.

The laboratory study of the N_2_O reduction path (in view of its field management) proved very positive since it validated the hypothesis that the factor explaining most of the variability of this path is soil pH, as this soil property can be managed for example by liming acidic soils with different materials^20^ and by preventing soil acidification^21^.

The role of soil pH in soil N_2_O emissions was previously reported in the literature^9,22^ and was mainly explained by the particularly high sensitivity of N_2_O reduction to low soil pH that prevents the reduction of N_2_O primarily by precluding the successful assembly of functional N_2_O reductase^23^. Due to its large sample size and the relevant distribution of soil samples properties, this current study further provides information indicating quantitative precision of the relationship between soil pH and the N_2_O reduction. First, even if the hypothesis of a linear relationship could not be rejected, a more complex pattern - as described by Fig. 2 - appeared, which concurs with the nonlinear relationship previously observed by ref. ^24^. Second, N_2_O reduction appeared inefficient at pH < 6.4 but was very efficient at pH > 6.8, with an intermediate zone of activation. These values are consistent with the laboratory experiment reported in ref. ^23^ and performed at pH values of 4.0, 6.1 and 8.0, during which the authors also observed that low soil pH prevented the reduction of N_2_O. Moreover, the targeted value of pH was observed to exceed 6.8. Recently, ref. ^22^ also highlighted the pH value of 6.8, based on the observation that the default EF (emission factor) value of 1.0% for agricultural soil proposed by the IPCC is relevant at this particular pH value and increases as pH decreases. From both the agronomic and environmental standpoints, a soil pH value of 6.8 appears to be reached with a reasonable input and by maintaining ecosystem equilibrium^21^. Recently, ref. ^25^ gained insight into the impact of liming on soil and crop processes together with functional aspects. As liming influences all elements in soils, ref. ^25^ highlighted the clear positive impacts of liming for crops by increasing P availability, decreasing the uptake of toxic heavy metals, and preserving and improving the physical properties of soils. Nevertheless, ref. ^25^ also noted that although there is a positive yield response for more crops, liming could also lead to the development of different crop diseases and deficiencies in trace-elements. Consequently, they proposed that liming management should be adjusted according to crop type within a given rotation. While ref. ^20^ reported that the target soil pH for cropped land in the UK is set at 6.5, this study suggests that raising it slightly to 6.8, while obviously taking into account sensitivity to crop disease, could greatly increase the environmental benefits of liming through N_2_O emission mitigation. Soil CEC also appeared to improve the prediction of the indicators of the capacity of soils to reduce N_2_O. The influence of CEC together with that of pH is consistent with the assumption that soil exchange capacity is one of the buffering mechanisms in soil^20^, sometimes taken into account in the calculation of soil liming requirements^21^. Concerning the influence of clay content observed, ref. ^9^ also observed consistently lower N_2_O emission for fine-textured soils. During the field experiments, we observed the abatement of N_2_O emissions due to liming, with a simultaneous increase in soil pH and the capacity of soil to reduce N_2_O, while the ancillary variables generally known to affect soil N_2_O emission (soil moisture, temperature and mineral nitrogen contents) were not affected by liming. The pH effect was observed to occur rapidly, in accordance with the mechanism of posttranscriptional interference of the expression of *nosZ*^23^. The abatement values we obtained (26%, 49% and 66%) appear reasonable, as a rough current estimation as ref. ^26^ also reported N_2_O emission abatement values from 16% to 64% due to liming and as a function of soil type, temperature, urine amendment and liming rate. Moreover, ref. ^27^ reported values between 60% and 73% after the application of dolomite to an acidic soil. It generally appears that abatement due to liming can be considerable at the field scale but varies with temperature, rainfall and nitrogen availability as well as the relative contribution of nitrification and denitrification in N_2_O production. In this study, the application of the NOE algorithm^28^ (Supplementary Information 5) to the database collected (Supplementary Dataset 1) suggested that (i) in E1, where N_2_O emissions were high, these emissions were mainly due to denitrification whereas (ii) in E2, where N_2_O emissions remained low, nitrification was the main process involved in them. Therefore, both the observed and simulated abatements obtained by managing soil pH and thus the soil N_2_O reduction path, (as relative and absolute values), were higher in E1 than in E2. While we were not able to definitively define how abatement can vary with fertilisation and rainfall, it seems that the abatement would be higher in situations where denitrification dominates nitrification, as the source of N_2_O. While these situations are expected to emit the highest N_2_O emissions, the abatement rate could increase with N_2_O emission rates. Nevertheless, this would require more investigations. The use of isotopic techniques to characterise the N_2_O/N_2_ ratio as well as the contribution of nitrification/denitrification to N_2_O emissions combined with a modelling approach in experimental situations appears to be a promising methodology for clarifying the combined effect of agronomic and environmental factors on the abatement rate.

Regarding the spatial up-scaling approach, all the relationships we defined, i.e., between soil N_2_O reduction and pH and between soil N_2_O reduction and pH, CEC and clay content (PTF functions), can be applied to all soils in France, regardless of their use (i.e. crops, grassland and forest) or characteristics. This is in agreement with the results obtained by ref. ^29^, which can be summarized as “the ratio of denitrification products is pH-specific rather than soil-specific”. Consistencies between our results and the literature^22–24^ suggest that the functions proposed are valid not only in France but perhaps also in different parts of the world (e.g., China). Nevertheless, this requires testing, primarily in countries with a soil monitoring network or with a soil properties database, for reasons of experimental facility.

As far as applicability (the relevant soil surface on which the solution can be applied) is concerned, consistent with the laboratory and field results obtained in this study, we defined this surface as an initial but necessary approach based solely on soil pH and/or the capacity to reduce N_2_O. We then calculated a high applicability value for France, but this value should be considered as a potential value. To go further with the liming solution for mitigating GHG emissions, several agronomic, environmental and socio-economic criteria would have to be introduced in the definition of applicability. Concerning agronomic criteria, further investigations are required to estimate surface areas vulnerable to the development of crop diseases attributable to increased soil pH and to deficiencies in trace elements^25^. Concerning environmental criteria, it will be essential to consider the impact of liming on other components of the greenhouse effect. An initial approach based on the current IPCC guideline^30^ suggests that enhanced CO_2_ emissions could roughly offset avoided N_2_O emissions in the case where all the C in the lime applied is emitted as CO_2_ in the year of application and that the duration of the effect of liming would be no longer than one year. Although the effect of liming on CO_2_ emissions is still subject to debate^31–33^ and the duration of the effect of liming is generally observed over several years^34,35^, we developed a preliminary calculation of the GHG balance obtained by liming. These calculations currently remain relatively global and do not consider the diversity of liming materials^20^ or specific soil requirements^21^. They are also quite uncertain due to perfectible knowledge on the fate of liming products in soils (ref. ^31^ suggested that CO_2_ emissions after liming may be less than half the maximum value due to the transport of dissolved inorganic C through rivers and lakes to the ocean), and on their effects on carbon (e.g., carbon storage^36^) and nitrogen cycles (currently the avoidance of N_2_O emissions). We then developed a methodology to calculate the GHG balance of liming (Supplementary Information 6). This methodology included (i) defining liming conditions (pH increase objectives, the need for a neutralizing value, type of product, frequency of application), (ii) defining CO_2_ emissions per application (during product preparation, during its transportation and application, during its presence in soils), (iii) defining greenhouse gas (GHG) benefits per application (soil organic storage^36,37^, avoided N_2_O emissions) and (iv) calculating the balance between CO_2_ emissions and GHG benefits. We then applied this methodology to several cases studies: (i) CaO and CaCO_3_ as liming materials, (ii) the requirement of 1000 and of 2500 neutralizing values (NV), and (iii) 2 hypotheses on the fate of liming products in soils (hypothesis that suggests that all C in liming products is transformed into CO_2_ in soils^30^ and hypothesis which considers that only half of the C in liming products is transformed into CO_2_^31^. We also introduced values from the scientific and technical literature (the CO_2_ emissions during product preparation kindly provided by the European Lime association, soil C storage^36^, the duration of the effect of liming products on soil pH^34,35^ and thus on soil N_2_O emissions). Whatever the product type, and for both hypothesis concerning the fate of carbonate in soils^30,31^, the GHG balance appeared positive for an application of 1000 NV ha^−1^. For an application as high as 2500 NV ha^−1^, the GHG balance appeared negative under the IPCC hypothesis for the two materials, but positive under the other hypothesis^31^ for carbonated products. Moreover, it should be noted that (i) national inventories currently explicitly consider CO_2_ emissions resulting from the application of lime, (ii) by-products exist with liming properties that could be utilised in certain situations^20^, (iii) very recently, ref. ^38^ suggested that the addition of crushed, fast reacting silicate rocks to croplands could also provide a combined strategy for CO_2_ removal and that (iv) N_2_O is also involved in stratospheric ozone depletion^1^.

Moreover, ref. ^39^ reported that mineral N fertilization induced acidification and disturbed N_2_O reduction, therefore resulting in increased N_2_O emissions. In addition to mineral fertilization, no-tillage can decrease soil pH^40^ and may therefore promote N_2_O emissions by slowing soil N_2_O reduction. The indirect effects of these practices on N_2_O reduction would also have to be considered in the future.

While liming as a solution could represent a significant cost for farmers, we also suggest expanding biotechnical research to encompass economic studies in the framework of ecosystemic climate regulation services, in view to sharing the cost of this possible multi-beneficiary mitigation action.

So far, although further biotechnical research is still required, especially in order not to create new sources of CO_2_ by decreasing N_2_O emissions, it seems that the agricultural sector, together with its stakeholders^41^, is capable of organising the combined application of solutions such as liming, N fertilization optimization, and using leguminous crops to decrease its GHG emissions. Finally, this study provided new and useful quantitative data (i) on the relationship between soil pH and the N_2_O reduction path, and (ii) on the potential of N_2_O emission mitigation at the plot and national scales by liming acidic soils and preventing soil acidification. The precautionary principle requires that the fate of the carbon added to soil when liming, as well as changes in sensitivity to crop disease should be investigated further before the large-scale deployment of this solution. We finally recommend that possible large-scale deployment should be based on local studies focusing on agronomic (local risk of disease and deficiencies), environmental (CO_2_/N_2_O balance) and economic aspects.

## Materials and Methods

### Laboratory determination of the capacity of soil to reduce N_2_O

The capacity of soil to reduce N_2_O was determined on selected samples from the French Soil Monitoring Network (RMQS)^10^, of which numerous biogeochemical properties are known (Supplementary Information S1_1). The selection was performed using the conditioned Latin hypercube sampling (cLHS) method^42^. This method is a stratified random procedure that provides an efficient way of sampling variables from their multivariate distributions. The aim of the sampling plan was to obtain samples distributed over the entire surface of the country, covering the national gradient of pH and organic carbon, and land-use. Specific soil samples were collected from the 0–27 (±4) cm soil layer from September 2012 to December 2015 according to the RMQS methodology^43^. Soil pH was also determined on 90 fresh RMQS samples, in accordance with ISO 10390:2005 (pH in H_2_O).

The capacity of soil to reduce N_2_O was determined in the laboratory according to ISO/TS 20131-2 derived from ref. ^8^. Four sets of incubation under anaerobic conditions were established using fresh soil samples sieved through a 5-mm mesh. Each set included three replicates equivalent to 50 g of dry soil placed in 565-ml flasks. Two series of incubations were carried out with the addition of NO_3_^−^ (50 ml KNO_3_ solution with 100 mg l^−1^), (1) in the presence of acetylene (2.5% of the gas atmosphere), and (2) without the addition of acetylene. These two series were used to determine the part of N_2_O emitted over time during the denitrification process. Two other series of incubations were carried out with the addition of water (50 ml) and N_2_O (5 ml) (1) in the presence of acetylene and (2) without acetylene. Due to reservations regarding the acetylene methodology^44^, the aim of these two series of incubations was to validate the total inhibition of N_2_O reductase by acetylene in our experimental conditions. Anaerobic conditions in the flasks were established before gas addition by three successive cycles of emptying and refilling with N_2_. The kinetics at 20 °C were determined over a period of one week with the flasks under constant agitation on a rotating shaker. The flasks were periodically sampled in pre-emptied flasks before analysis on a µGC (SRA Instruments T3000) equipped with a TCD detector. The results of this test could be represented in a graph of the quantity of N_2_O produced in flasks over time for the two treatments with nitrate addition and synthetized through two indicators, r_max_ and index. r_max_ is the maximum ratio of the accumulated N_2_O during incubation, with r being the ratio between N_2_O produced during incubation without acetylene to N_2_O produced during incubation with acetylene, at each time point. The index combines information on the level of r_max_ and on the time of accumulation of N_2_O during incubation. The higher the r_max_ and index are, the lower the capacity of soil to reduce N_2_O. Supplementary Information 2 illustrates this method for three soils studied in this study. ISO/TS 20131-2, annex A, indicates that soils with an index higher than 50 are especially suspected to emit very high levels of N_2_O due to low N_2_O reduction activity. An index higher than 50 generally corresponds either to an absence of N_2_O reduction (r ≥ 1) over a period of at least 48 hours or to a very poor reduction of N_2_O (r ~ 0.8) over a period of at least 72 h. We generally consider that soils are poorly able to reduce N_2_O when the index is higher than 50 or when r_max_ is higher than 0.8. Soils are very effective at reducing N_2_O when the r_max_ and index are lower than 0.4 and 30, respectively.

The statistical methods applied to the obtained database mainly include linear regression and partial least square regression (PLS)^12^, generalized boosted models (GBMs)^13^ and classification and regression trees (CART)^14^ coupled to factorial discriminant analysis (FDA)^15^. CART and FDA were applied to 98% of the studied RMQS subset, using index values related to soil pH to define pH-index classes.

### Field experiment for soil N_2_O emission measurement

In addition to the laboratory tests, two experimental block trials (E1 and E2) were performed at the field scale. They involved cultivated fields managed by French technical Institutes, with soils that were initially acidic and hardly able to reduce N_2_O. E1 was dedicated to the effect of liming and E2 to the effect of liming and the addition of organic matter.

The E1 experiment was conducted (initial pH < 6.0) on the Arvalis-Institut du Végétal domain of La Jaillière (France, 57.451°N, 0.966°W) in 2013–2014 and 2014–2015. The soil at this experimental site is composed of gleyic cambisols lying on an alterite consisting of sandstony schist, widely observed in the West of France. The texture class of the arable soil is ‘loamy silty sand’ with a carbon content of 1.3%. The climate at this site is of oceanic type with an average temperature of 12.5 °C and a mean annual precipitation of 730 mm. The site is generally cultivated with a succession of Maize, Maize, Wheat. During this experiment, E1 was successively cultivated with fertilized maize and wheat. pH management consisted of the addition of 1 t CaO ha^−1^ in September 2013 and in September 2014. During the first year of the experiment, nitrogen fertiliser was spread once at the 3-leaf stage on 14/05/2014, with 60 kg of N ha^−1^ (33.5% ammonium nitrate). During the second year of the experiment, three inputs of nitrogen were spread in spring using ammonium nitrate at 33.5%: 70 kg of N ha^−1^ on 09/03/2015, then 50 kg of N ha^−1^ on 30/03/2015, and lastly 60 kg of N ha^−1^ on 10/05/2015.

The E2 experiment was conducted (initial pH < 5.6), on a field managed by Terres Inovia at Presly la Noue (France, 47.388°N, 2.357°E) in 2013–2014. The soil was podzolic, the texture class of the arable field was ‘sandy loamy’ and the carbon content was 1.9%. The climate at the site is of oceanic type with an average temperature of 10.7 °C and a mean annual precipitation of 710 mm. Prior to the experiment the soil of E2 was kept bare. During E2, the soil was cultivated with fertilized mustard. pH management consisted of the addition of 1.5 t CaO ha^−1^ to E2. CaO was added at the time of implantation, with burial immediately after input at a depth of 10 cm. Soil organic matter management consisted of the addition of 27 m^3^ ha^−1^ y^−1^ of pork liquid manure. The lime and manure were spread one week before seeding the crop (winter rapeseed) so as not to risk damaging the seed and jeopardising emergence. The pig manure was spread at a rate of 25 m^3^ ha^−1^. Mineral fertilisers were spread during two events: the first input was 50 kg N ha^−1^ on 2/05/2014 and the second was 50 kg N ha^−1^ on 9/05/2014. Only the “pig manure” plots did not receive the second input. Additional agricultural technical processes are described in Supplementary Information 7.

Field sites were organized in random blocks with three blocks per treatment; each block consisted of three chambers. *In situ* N_2_O emission measurements were performed using the manual chamber method coupled with gas chromatography (electron capture detector) analysis^45,46^. All the N_2_O measurements also included measurements of the ancillary variables (soil moisture, mineral nitrogen and temperature). Soil moisture and mineral nitrogen determinations involved 1 replicate per block (i.e. 3 replicates per treatment), each obtained from a composite of 10 soil samples collected with an auger. The pH and capacity to reduce N_2_O were determined periodically on these soil samples. Soil temperature was directly measured in the field.

The Wilcoxon signed rank test was used site per site to test the null hypothesis that the treatments (liming and organic matter addition in comparison with control) did not affect soil N_2_O emission over each annual period of measurements (p < 0.05) and did not affect soil ancillary variables (p < 0.05). In the case where this hypothesis was rejected, we used student’s t-test to determine the dates with significant differences (p < 0.05). The combined effect of ancillary variables was analysed using the NOE algorithm^28^. Except, the specific r_max_ values to each situation, all the other biological parameters required by NOE were measured on E1. The Wilcoxon signed rank test was used site per site to test the null hypothesis that the dynamics of measures and simulations were not different (p < 0.05). We also used the RMSE criteria to evaluate the quality of the simulations.

### Upscaling to the national scale

We used different approaches (“CART-FDA”, “cumulative frequency”, “map_rmqs”) for calculating the potential applicability of soil liming for reducing N_2_O. The use of different relevant approaches allows for the proposal of an interval of estimated values.

As far as the “CART-FDA” approach is concerned, the previously defined classes of index-pH were applied to the soil pH of the total RMQS to assess the index frequency of cultivated soils and of fertilized grassland soils at the national scale. Thresholding consisted of evaluating the surface of soils in the different classes of pH-index.

During the “cumulative frequency approach”, we determined the cumulative frequencies of soil pH, soil r_max_ and soil index. We assessed the soil r_max_ and soil index by applying Eqs. 1 and 2 to the entire RMQS database. For pH as phenotype indicator, thresholding was applied to quantify the surface of soils having a pH lower than 6.8 ([soil] _PhN2Ored+/− and PhN2Ored−_), soils having a pH lower than 6.4 ([soil]_PhN2Ored−_). For r_max_, in accordance with both ISO/TS20141–2 and the GBM function, thresholding was applied to quantify the surface of soils having a predicted r_max_ higher than 0.4 ([soil] _PhN2Ored+/− and PhN2Ored−_) and especially the surface of soils having a r_max_ higher than 0.8 ([soil]_PhN2Ored−_). Concerning the index, thresholding was applied to quantify the surface of soils having a predicted index value higher than 30 ([soil] _PhN2Ored+/− and PhN2Ored−_) and especially higher than 50 ([soil]_PhN2Ored−_). Finally, we estimated the surface of soils for each phenotype as the median surface obtained using each indicator.

**Figure 5:**
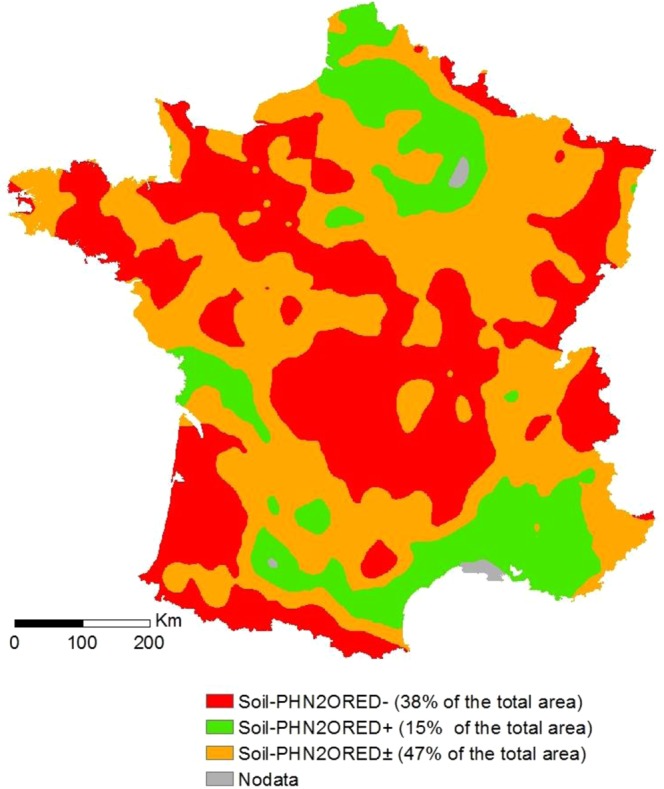
Map of soil N_2_O reduction phenotypes in France obtained from the RMQS database.

As far as the “map_rmqs approach” was concerned, we first mapped soil pH, CEC and the clay content by kriging. We then inferred the r_max_ map over the whole of France’s continental area using Eq. 1 (Fig. 5A). A thresholding with r_max_ ≥ 0.8 corresponding to [soil]_PhN2Ored−_, r_max_ < 0.4 corresponding to [soil]_PhN2Ored+,_ and 0.4 ≤ r_max_ < 0.8 corresponding to [soil]_PhN2Ored+/−_ was then applied to define the potential applicability of liming to reduce N_2_O emissions at the national scale.

## Supplementary information

SI_1.

SI_2.

SI_3.

SI_4.

SI_5.

SI_6.

SI_7.

Supplementary Dataset 1.

## Data Availability

Data are available on request from catherine.henault@inra.fr.
