# Supplementary material for: Management of soil pH promotes nitrous oxide reduction and thus mitigates soil emissions of this greenhouse gas
Source: Sci Rep. 2019 Dec 27;9:20182. doi: 10.1038/s41598-019-56694-3 (PMC6934481; doi:10.1038/s41598-019-56694-3)
Supplement: Supplementary file 1 — SI_1. [file 41598_2019_56694_MOESM1_ESM.pdf]

Management of soil pH promotes nitrous oxide reduction and thus mitigates soil emissions of this greenhouse gas

Catherine Hénault[1,2 (*)], Hocine Bourennane[2], Adeline Ayzac[2], Céline Ratié[3], Nicolas Saby[3], Jean-Pierre Cohan[4], Thomas Eglin[5], Cécile Le Gall[6]

[1] Agroécologie, AgroSup Dijon, INRA, Univ. Bourgogne Franche-Comté, F-21000 Dijon, France

[2] URSOLS, INRA, 45075 Orléans, France

[3] Infosol, INRA, 45075 Orléans, France

[4]ARVALIS- Institut du Végétal Route de Châteaufort – RD 36 – ZA des Graviers

91190 – Villiers le Bacle, France

[5] ADEME, Direction Productions et Energies Durables, Service Forêts, Alimentation et Bioéconomie, F-49000 Angers, France

[6] TERRES INOVIA, Avenue Lucien Brétignières, 78850 Thiverval Grignon, France

Supplementary information 1: Informations dealing with the French Soil Monitoring Network (RMQS)

SI_1.1 : Soil properties routinely analysed during the RMQS program. Used methods are indicated in Ref[10]

- Particule size (without decarbonatation) 5 fractions
- Soil moisture
- pH in water
- Total calcareous
- Organic carbon after decarbonatation
- Total Nitrogen
- Available P
- CEC
- Exchangeable cations : Al, Ca, Fe, K, Mg, Mn, Na
- Total Major Elements : Al, Ca, Fe, K, Mg, Mn, Na
- Available Fe
- Bo in water
- Total Trace elements : Cr, Cu, Ni, Zn, Cd, Co, Mo, Pb, Tl
- Extractable trace elements : Cd, Cr, Cu, Ni, Pb, Zn

SI_1.2 : Comparison of the distribution of soil parameters ((a) soil carbon content, (b) soil pH, (c) land use and (d) geographical position) in the total RMQS and in the subset used in this study

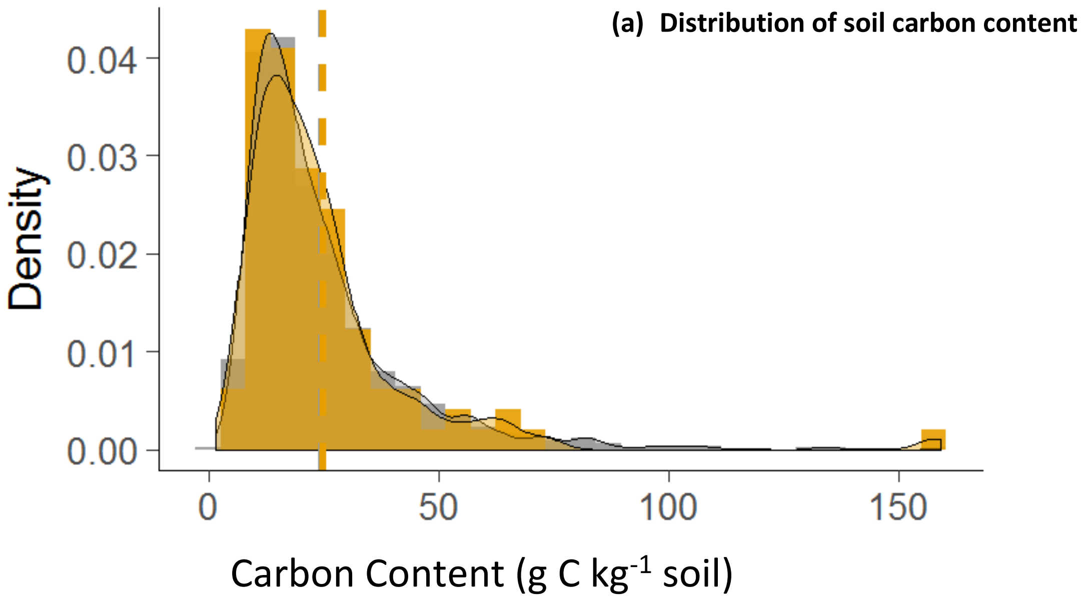

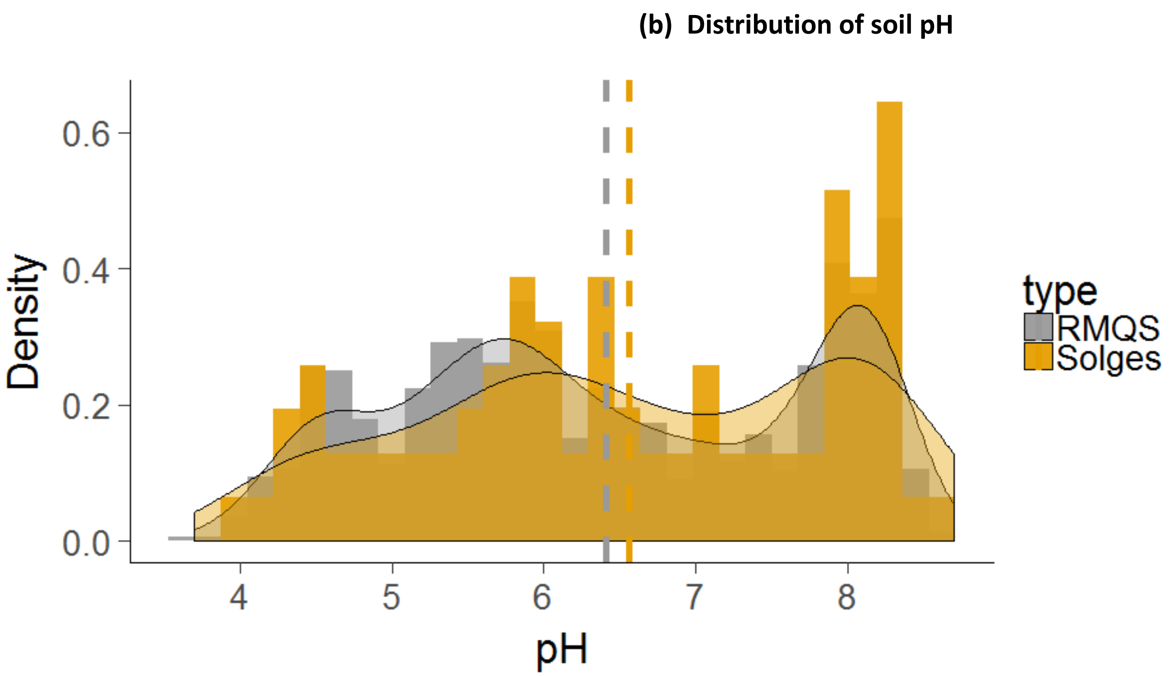

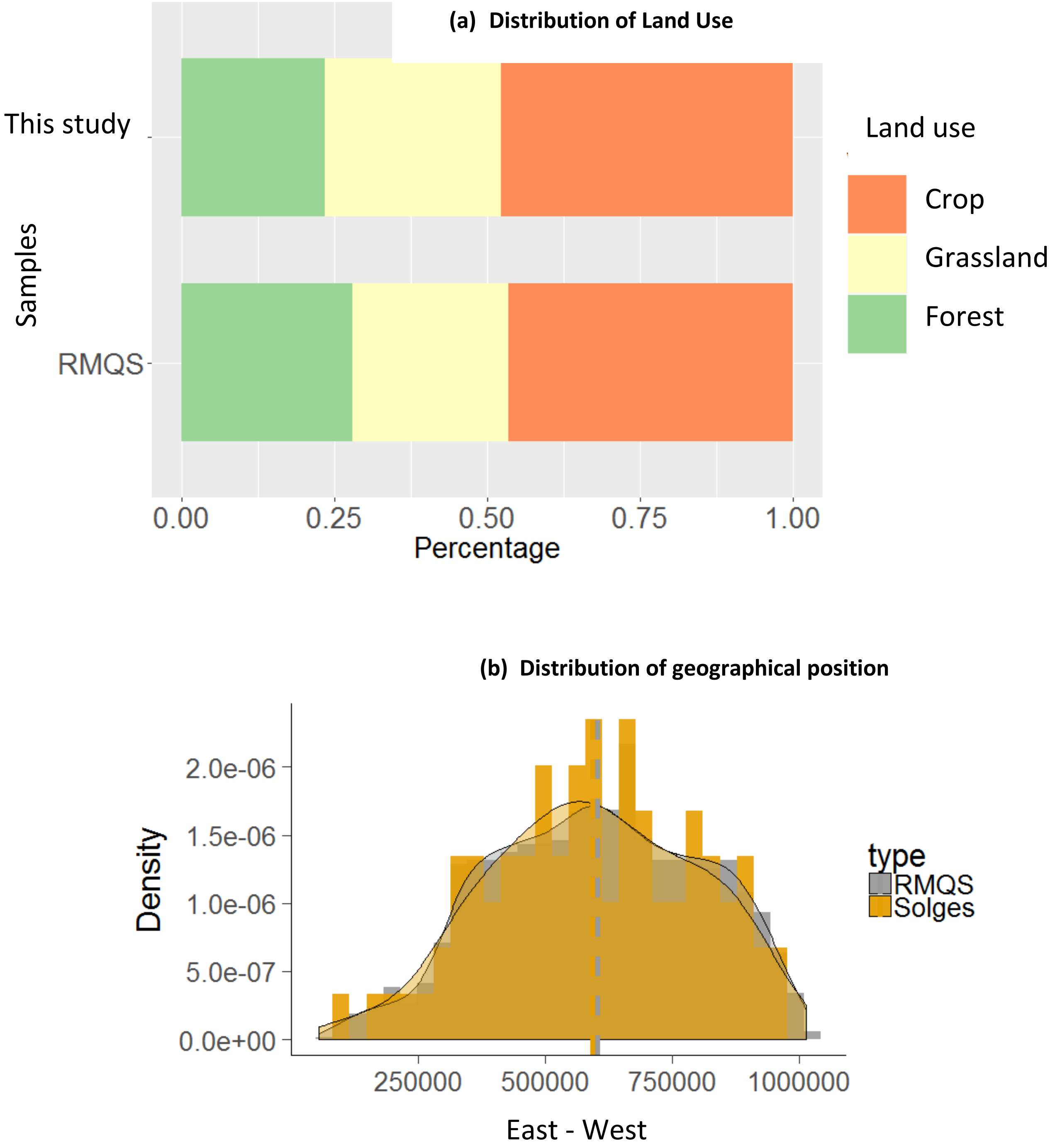

Figure SI_1.2 : Distribution of soil parameters
