# Supplementary material for: Management of soil pH promotes nitrous oxide reduction and thus mitigates soil emissions of this greenhouse gas
Source: Sci Rep. 2019 Dec 27;9:20182. doi: 10.1038/s41598-019-56694-3 (PMC6934481; doi:10.1038/s41598-019-56694-3)
Supplement: Supplementary file 2 — SI_2. [file 41598_2019_56694_MOESM2_ESM.pdf]

Management of soil pH promotes nitrous oxide reduction and thus mitigates soil emissions of this greenhouse gas

Catherine Hénault[1,2 (*)], Hocine Bourennane[2], Adeline Ayzac[2], Céline Ratié[3], Nicolas Saby[3], Jean-Pierre Cohan[4], Thomas Eglin[5], Cécile Le Gall[6]

[1] Agroécologie, AgroSup Dijon, INRA, Univ. Bourgogne Franche-Comté, F-21000 Dijon, France

[2] URSOLS, INRA, 45075 Orléans, France

[3] Infosol, INRA, 45075 Orléans, France

[4] ARVALIS- Institut du Végétal Route de Châteaufort – RD 36 – ZA des Graviers

91190 – Villiers le Bacle, France

[5] ADEME, Direction Productions et Energies Durables, Service Forêts, Alimentation et Bioéconomie, F-49000 Angers, France

[6] TERRES INOVIA, Avenue Lucien Brétignières, 78850 Thiverval Grignon, France

Supplementary information 2: Calculation of the r_{max} and index indicators for 3 studied soils, using results obtained during the laboratory experiment (protocol ISO/TS 20131-2, Ref[8])

mean N-N_2O Incubation with (NO_3^-)	mean N-N_2O Incubation with (NO_3^- +C_2H_2)	RATIO r	r $_{max}$	Accumulation max	time of accumulation (h)	INDEX
(µg N-N2O g^{-1} dry soil)						
0.23	0.55		1.02			**99.33**
5.56	6.23	0.8923				
14.58	14.24	1.0240				
30.95	33.33	0.9286				
67.42	78.96	0.8539		67.42	97	
0.30	122.89	0.0024				
0.21	0.52		0.64			**30.66**
4.77	7.46	0.6388				
5.56	14.93	0.3726		5.56	48	
5.07	21.46	0.2363				
4.52	27.13	0.1667				
3.44	39.08	0.0881				
0.28	0.45		0.06			**1.48**
0.79	12.81	0.0619		0.79	24	
0.00	40.35	0.0000				
0.08	63.97	0.0012				
0.20	82.27	0.0024				
0.22	101.08	0.0022				

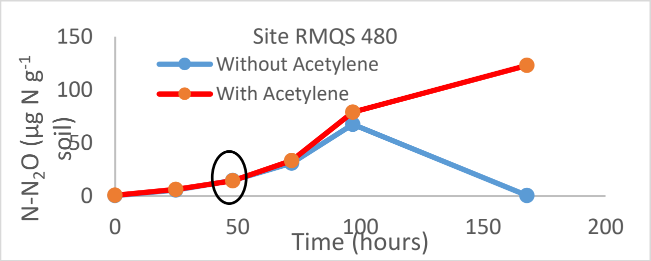

r_{max} (1.02) was observed 48 hours after the start of incubation

The time of accumulation was of 97 hours

The index value is 99

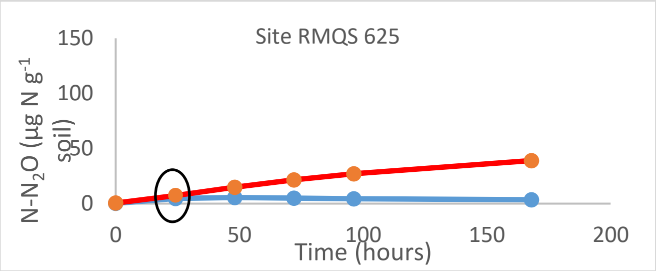

r_{max} (0.64) was observed 24 hours after the start of incubation

The time of accumulation was of 48 hours

The index value is 31

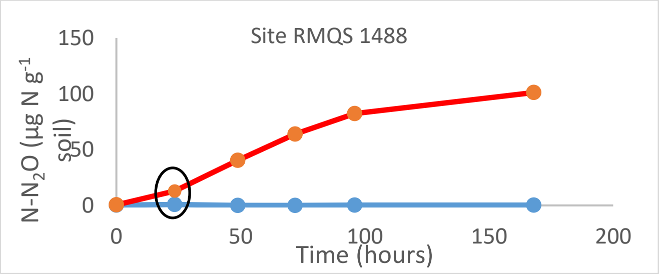

r_{max} (0.06) was observed 24 hours after the start of incubation

The time of accumulation was of 24 hours

The index value is 1
