# Supplementary material for: Management of soil pH promotes nitrous oxide reduction and thus mitigates soil emissions of this greenhouse gas
Source: Sci Rep. 2019 Dec 27;9:20182. doi: 10.1038/s41598-019-56694-3 (PMC6934481; doi:10.1038/s41598-019-56694-3)
Supplement: Supplementary file 3 — SI_3. [file 41598_2019_56694_MOESM3_ESM.pdf]

Management of soil pH promotes nitrous oxide reduction and thus mitigates soil emissions of this greenhouse gas

Catherine Hénault[1,2 (*)], Hocine Bourennane[2], Adeline Ayzac[2], Céline Ratié[3], Nicolas Saby[3], Jean-Pierre Cohan[4], Thomas Eglin[5], Cécile Le Gall[6]

[1] Agroécologie, AgroSup Dijon, INRA, Univ. Bourgogne Franche-Comté, F-21000 Dijon, France

[2] URSOLS, INRA, 45075 Orléans, France

[3] Infosol, INRA, 45075 Orléans, France

[4]ARVALIS- Institut du Végétal Route de Châteaufort – RD 36 – ZA des Graviers

91190 – Villiers le Bacle, France

[5] ADEME, Direction Productions et Energies Durables, Service Forêts, Alimentation et Bioéconomie, F-49000 Angers, France

[6] TERRES INOVIA, Avenue Lucien Brétignières, 78850 Thiverval Grignon, France

Supplementary information 3: Database of the main soil properties including information on their capacity to reduce N_2O

RMQS site number	Land Use	pH_{water}	C content ‰	r_{max}	index	PHN2ORED
10	Crop	6.43	11.9	0.7	117	-
27	Crop	7.83	17.1	0.35	17	+
53	Grassland	7.97	44	0.37	18	+
63	Grassland	8.09	28.6	0.52	25	±
158	Crop	7.76	12.9	0.27	13	+
211	Crop	8.22	20.6	0.44	21	±
215	Forest	7.61	54.9	0.47	11	±
231	Crop	6.99	14.2	0.34	16	+
244	Crop	7.24	10.4	0.79	38	±
280	Forest	4.2	16.6	1.1	210	-
312	Crop	7.05	14.6	0.01	0	+
316	Crop	6.65	14.6	0.58	27	±
352	Grassland	6.79	36.1	0.38	8	+
394	Crop	6.41	8.9	0.69	120	-
413	Grassland	7.93	24.8	0.44	21	±
445	Crop	6.35	15.4	0.8	39	±
456	Crop	7.75	10.7	0.45	43	±
480	Grassland	5.36	32.6	1.02	99	-
484	Grassland	5.49	45.8	1.11	107	-
519	Crop	8.38	12.8	0.24	6	+
554	Crop	6.37	27.7	0.72	34	±
590	Grassland	5.58	27.9	0.9	64	-
619	Crop	8.25	14.2	0.31	15	+
620	Forest	5.13	17.5	1.1	185	-
621	Crop	6.76	20.5	0.39	9	+
625	Crop	6.54	12.7	0.64	31	±
680	Crop	6.81	14.5	0.41	10	±
710	Forest	4.43	44.1	0.98	164	-
750	Crop	7.99	26	0.02	1	+
758	Grassland	7.54	25.1	0.25	6	+
763	Grassland	5.97	19.8	0.74	123	-
788	Grassland	6.24	14.6	0.74	71	-
805	Forest	4.45	13.4	0.55	93	-
829	Crop	6.59	26.1	0.52	13	±
831	Crop	6.11	19.1	0.69	16	±
835	Crop	5.86	14.7	0.83	60	-
839	Crop	6.25	9.39	1.13	186	-
917	Crop	7.31	20.5	0.67	50	±
936	Crop	6.19	19.9	0.83	40	-
940	Grassland	6.41	12.7	0.89	65	-
1017	Forest	5.75	46.1	0.97	94	-
1038	Crop	7.44	10.1	0.09	2	+
1084	Crop	6.37	9.29	0.7	117	-
1085	Crop	8.1	17.3	0.44	21	±
1087	Crop	8	21.1	0.33	8	+

1119	Forest	4.71	15.2	1.2	202	-
1142	Grassland	5.32	50.3	0.99	95	-
1163	Crop	8.09	25.6	0.27	6	+
1168	Grassland	7.04	8.37	0.69	33	±
1231	Forest	7.1	73.2	0.58	28	±
1245	Forest	6.38	23.1	0.68	66	-
1306	Grassland	5.63	36.7	0.79	40	±
1343	Forest	7.71	34.2	0.39	10	+
1353	Grassland	8.06	42.6	0.56	27	±
1355	Crop	7.16	17.6	0.22	5	+
1368	Crop	8.11	22.8	0.42	10	±
1413	Forest	5.85	17.5	1.2	202	-
1418	Grassland	5.8	28.9	0.97	92	-
1450	Crop	8.11	25.3	0.27	6	+
1488	Crop	6.87	8.35	0.06	1	+
1514	Forest	4.65	17.6	1.2	202	-
1559	Crop	6.69	18.9	0.35	8	+
1577	Grassland	8.17	20.6	0.36	18	+
1588	Grassland	6.08	58.4	0.83	60	-
1627	Forest	4.27	47.7	1.07	177	-
1653	Forest	4.71	13.7	1.2	202	-
1689	Forest	7.77	45.5	0.62	27	±
1695	Forest	4.27	41.5	0.55	90	-
1717	Crop	5.33	23	0.81	130	-
1756	Forest	4.94	158	1.07	102	-
1803	Forest	4.86	29.8	1.2	202	-
1835	Grassland	7.71	30	0.47	22	±
1841	Grassland	7.62	59.6	0.63	17	±
1881	Crop	6.27	19.1	0.89	42	-
1917	Crop	8.31	10.4	0.18	5	+
1936	Forest	8.07	28.4	0.12	3	+
1970	Crop	6.29	11.1	1.02	166	-
1986	Crop	8.32	11.4	0.23	6	+
2002	Forest	4.71	20	0.7	113	-
2062	Crop	8.47	10.4	0.18	4	+
2123	Grassland	8.21	12.8	0.19	32	±
2128	Crop	8.5	8.18	0.14	14	+
2138	Grassland	6.99	30.2	0.55	41	±
2188	Grassland	8.21	37.3	0.27	7	+
2191	Grassland	7.31	13.9	0.94	152	-
2224	Forest	6.18	52.4	0.31	8	+
2319	Crop	6.18	9.34	0.93	149	-
10289	Grassland	5.88	23.9	0.91	170	-
10967	Crop	8.14	23.3	0.24	7	+
11797	Grassland	8.23	20.2	0.02	0	+
