# Supplementary material for: Management of soil pH promotes nitrous oxide reduction and thus mitigates soil emissions of this greenhouse gas
Source: Sci Rep. 2019 Dec 27;9:20182. doi: 10.1038/s41598-019-56694-3 (PMC6934481; doi:10.1038/s41598-019-56694-3)
Supplement: Supplementary file 4 — SI_4. [file 41598_2019_56694_MOESM4_ESM.pdf]

Management of soil pH promotes nitrous oxide reduction and thus mitigates soil emissions of this greenhouse gas

Catherine Hénault[1,2 (*)], Hocine Bourennane[2], Adeline Ayzac[2], Céline Ratié[3], Nicolas Saby[3], Jean-Pierre Cohan[4], Thomas Eglin[5], Cécile Le Gall[6]

[1] Agroécologie, AgroSup Dijon, INRA, Univ. Bourgogne Franche-Comté, F-21000 Dijon, France

[2] URSOLS, INRA, 45075 Orléans, France

[3] Infosol, INRA, 45075 Orléans, France

[4]ARVALIS- Institut du Végétal Route de Châteaufort – RD 36 – ZA des Graviers

91190 – Villiers le Bacle, France

[5] ADEME, Direction Productions et Energies Durables, Service Forêts, Alimentation et Bioéconomie, F-49000 Angers, France

[6] TERRES INOVIA, Avenue Lucien Brétignières, 78850 Thiverval Grignon, France

Supplementary information 4: Potential applicability of soil liming for reducing N_2O

SI4_1. "The CART-FDA approach"

Table SI4_1 : Classification rules defined for the CART methodology on the RMQS subset

Number of soil samples (representativity in the total sample of 88 soils)	Rule
6 (7%)	When pH is in the range [3.96 ; 4.54], index = 173 for 7% of soils
7 (8%)	When pH is in the range [4.54 ; 5.16], index = 172 for 8 % of soils
8 (9%)	When pH is in the range [5.16 ; 5.72], index = 115 for 9% of soils
17 (19%)	When pH is in the range [5.72 ; 6.38], index = 95 for 19% of soils
16 (18%)	When pH is in the range [6.38 ; 7.06], index = 26.4 for 18% of soils
5 (6%)	When pH is in the range [7.06 ; 7.67], index = 11 for 6% of soils
18 (20%)	When pH is in the range [7.67; 8.2], index = 15 for 20% of soils
11 (13%)	When pH is in the range [8.2 ; 9.7], index = 13 for 13% of soils

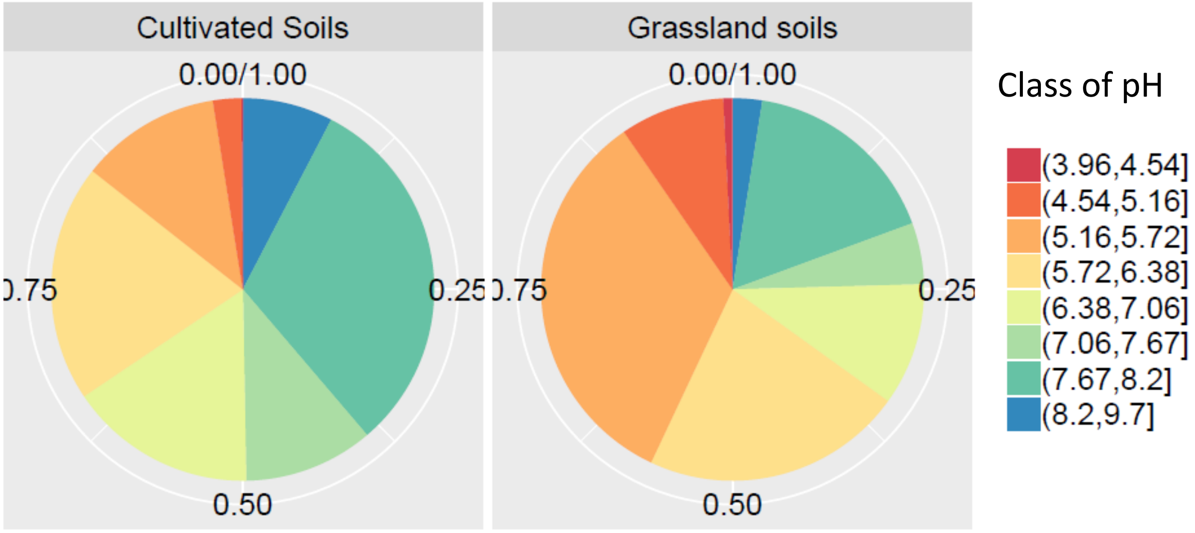

Figure SI4_1: Estimation of the contribution of each class of pH at the national scale (France) on the basis of the RMQS database

The contribution of cultivated soils and grassland soils having a pH < 6.4 and then an index > 50 was estimated to 35 % and 65 % respectively leading to a potential applicability of 0.45 (= 0.68*0.35+0.32*0.62) . The class of pH comprised between 6.4 and 6.8 being included in the larger class of pH comprised between 6.38 and 7.06 was not taken into account in the potential applicability assessment in order to avoid overestimation.

SI_4.2 The "cumulative frequency approach"

Table SI4-21 : Cumulative frequency of soil pH, obtained from the RMQS, and of the indicators r_{max} and index obtained using Equations 1 and 2, at the national scale in France. The thresholding step is represented by coloured cells in the table.

	Cultivated soils			Grassland soils			
Cumulative frequencies	pH	r_{max}	index	Cumulative frequencies	pH	r_{max}	index
0	4.5	0	0	0	4.5	0	0
5%	5.3	0	0	5%	5	0.13	0
10%	5.5	0.01	0	10%	5.2	0.25	0
15%	5.7	0.07	0	15%	5.3	0.35	19
20%	5.9	0.11	0	20%	5.4	0.47	45
25%	6.1	0.17	0	25%	5.5	0.6	70
30%	6.2	0.21	0	30%	5.6	0.67	84
35%	6.4	0.26	0	35%	5.62	0.73	94
40%	6.6	0.33	11	40%	5.7	0.77	102
45%	6.8	0.39	20	45%	5.8	0.81	111
50%	7	0.44	32	50%	5.9	0.84	119
55%	7.2	0.51	48	55%	6	0.88	124
60%	7.6	0.58	60	60%	6.11	0.9	129
65%	7.8	0.64	70	65%	6.4	0.92	135
70%	8	0.7	83	70%	6.64	0.95	141
75%	8.1	0.74	94	75%	6.94	0.97	148
80%	8.1	0.8	104	80%	7.5	0.99	156
85%	8.2	0.85	118	85%	7.84	1.02	165
90%	8.2	0.91	132	90%	8	1.06	174
95%	8.3	1	153	95%	8.1	1.11	190
100%	8.6	1.2	202	100%	8.63	1.2	202

$[soils]_{Phred+/-}$	$[soils]_{Phred-}$	$[soils]_{Phred+}$

Table SI4-22 : Estimation of soil surfaces with the phenotypes obtained by calculating the median result for each indicator. This calculation are made at the France scale.

			Rules	Part of surface
Cultivated soils (68 % of the fertilised soils)				
Estimation with	pH	$[Soils]_{Phred-}$	pH ≤ 6.4	35%
		$[Soils]_{Phred-}$ and $[soils]_{Phred+/-}$	pH ≤ 6.8	45%
	r_{max}	$[Soils]_{Phred-}$	r_{max} ≥ 0.8	20%
		$[Soils]_{Phred-}$ and $[soils]_{Phred+/-}$	r_{max} ≥ 0. 4	50%
	Index	$[Soils]_{Phred-}$	index ≥ 50	40%
		$[Soils]_{Phred-}$ and $[soils]_{Phred+/-}$	index ≥ 30	50%
Grassland soils (32 % of fertilised soils)				
Estimation with	pH	$[Soils]_{Phred-}$	pH ≤ 6.4	65%
		$[Soils]_{Phred-}$ and $[soils]_{Phred+/-}$	pH ≤ 6.8	70%
	r_{max}	$[Soils]_{Phred-}$	r_{max} ≥ 0.8	55%
		$[Soils]_{Phred-}$ and $[soils]_{Phred+/-}$	r_{max} ≥ 0. 4	80%
	Index	$[Soils]_{Phred-}$	index ≥ 50	75%
		$[Soils]_{Phred-}$ and $[soils]_{Phred+/-}$	index ≥ 30	80%
All fertilised soils (weighted)				
Estimation with	pH, r_{max} and index	$[Soils]_{Phred-}$		45%
		$[Soils]_{Phred-}$ and $[soils]_{Phred+/-}$		60%
		$[Soils]_{Phred+/-}$		15%

45 % = 0.68*median(35%;25%;45%)+0.32*median(65%;60%;.80%)
60% = 0.68*median(45%;50%;50%)+0.32*medain(70%;80%;80%)

SI_4.3. The "map_rmqs approach"

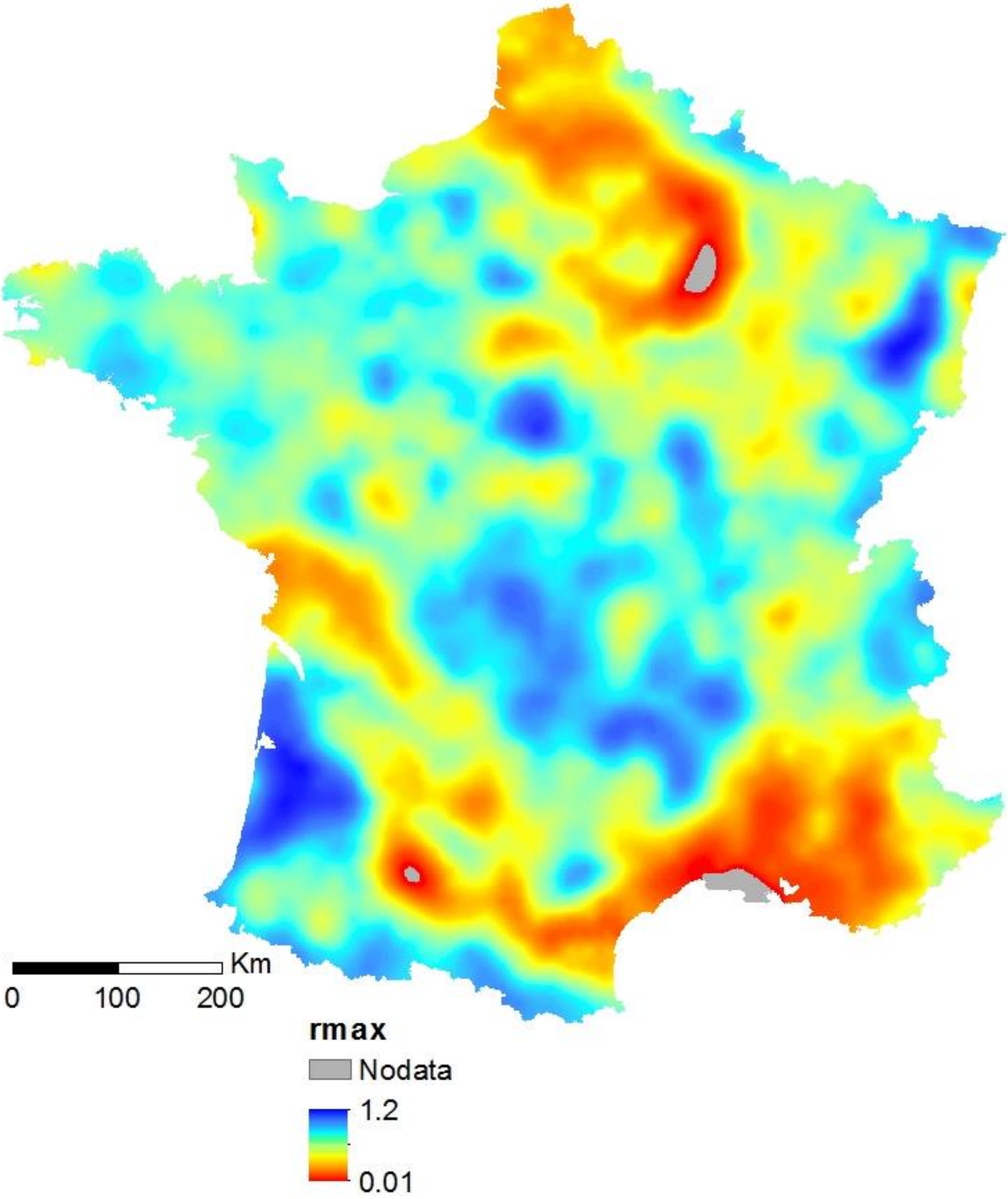

Figure SI_4.3. Map of r_{max} obtained by inference of Equation 1 after applying kriging to soil pH, CEC and the clay content
