# Supplementary material for: Management of soil pH promotes nitrous oxide reduction and thus mitigates soil emissions of this greenhouse gas
Source: Sci Rep. 2019 Dec 27;9:20182. doi: 10.1038/s41598-019-56694-3 (PMC6934481; doi:10.1038/s41598-019-56694-3)
Supplement: Supplementary file 5 — SI_5. [file 41598_2019_56694_MOESM5_ESM.pdf]

Management of soil pH promotes nitrous oxide reduction and thus mitigates soil emissions of this greenhouse gas

Catherine Hénault[1,2 (*)], Hocine Bourennane[2], Adeline Ayzac[2], Céline Ratié[3], Nicolas Saby[3], Jean-Pierre Cohan[4], Thomas Eglin[5], Cécile Le Gall[6]

[1] Agroécologie, AgroSup Dijon, INRA, Univ. Bourgogne Franche-Comté, F-21000 Dijon, France

[2] URSOLS, INRA, 45075 Orléans, France

[3] Infosol, INRA, 45075 Orléans, France

[4]ARVALIS- Institut du Végétal Route de Châteaufort – RD 36 – ZA des Graviers

91190 – Villiers le Bacle, France

[5] ADEME, Direction Productions et Energies Durables, Service Forêts, Alimentation et Bioéconomie, F-49000 Angers, France

[6] TERRES INOVIA, Avenue Lucien Brétignières, 78850 Thiverval Grignon, France

Supplementary information 5 : Application of the NOE algorithm to the field database dealing with soil N_2O fluxes

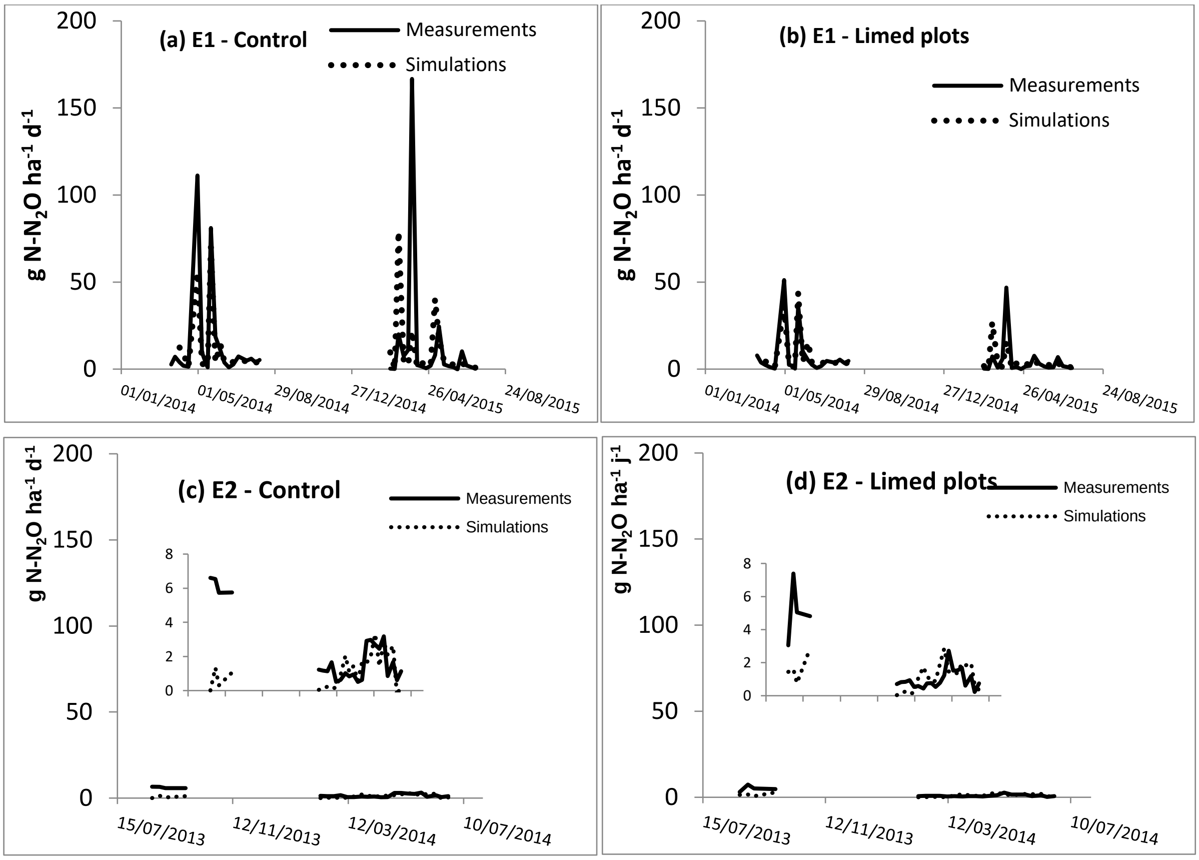

Figure SI_5 : Comparison of measured and simulated N_2O fluxes in the E1 and E2 experiment. Due to the very low fluxes in E2 compared to those obtained in E1, E2 figures included an extended representation. Measured and simulated dynamics of N_2O fluxes were not significantly different in all situations and RMSE values always appeared quite low.
