# Supplementary material for: Management of soil pH promotes nitrous oxide reduction and thus mitigates soil emissions of this greenhouse gas
Source: Sci Rep. 2019 Dec 27;9:20182. doi: 10.1038/s41598-019-56694-3 (PMC6934481; doi:10.1038/s41598-019-56694-3)
Supplement: Supplementary file 6 — SI_6. [file 41598_2019_56694_MOESM6_ESM.pdf]

Management of soil pH promotes nitrous oxide reduction and thus mitigates soil emissions of this greenhouse gas

Catherine Hénault[1,2 (*)], Hocine Bourennane[2], Adeline Ayzac[2], Céline Ratié[3], Nicolas Saby[3], Jean-Pierre Cohan[4], Thomas Eglin[5], Cécile Le Gall[6]

[1] Agroécologie, AgroSup Dijon, INRA, Univ. Bourgogne Franche-Comté, F-21000 Dijon, France

[2] URSOLS, INRA, 45075 Orléans, France

[3] Infosol, INRA, 45075 Orléans, France

[4]ARVALIS- Institut du Végétal Route de Châteaufort – RD 36 – ZA des Graviers

91190 – Villiers le Bacle, France

[5] ADEME, Direction Productions et Energies Durables, Service Forêts, Alimentation et Bioéconomie, F-49000 Angers, France

[6] TERRES INOVIA, Avenue Lucien Brétignières, 78850 Thiverval Grignon, France

Supplementary Information 6 : Calculation of the GHG balance of liming action

	Variable	Hypothesis	unit	Value				Comments / Source
Description of liming conditions	Type of liming			Liming (for a pH increase of around 0.5 point)		Liming (for a pH increase higher than 1 point)		
	Requirement of neutralizing value		VN ha^{-1}	1000	1000	2500	2500	
	Product			CaO	CaCO3	CaO	CaCO3	
	Neutralizing value (%)		%	92	52	92	52	
	Requirement of product		kg ha^{-1}	1087	1923	2717	4808	
	Frequency of Application (years)		years	4	4	5	5	https://comifer.asso.fr/images/publications/brochures/brochure_chaulage%20maj%202012_chaulage%20lt.pdf
CO2 emissions per application at a 4 years frequency	Product preparation	generic value	kg CO_2 kg^{-1}	1.132	0.040	1.132	0.040	Data Source : EuLA, the European Lime Association
			kg CO_2 ha^{-1}	1230	77	3076	192	
	Transport and application	generic value	kg CO2 kg-1 product	0.005	0.005	0.005	0.005	basic calculation with a fuel consumption of 35 l per 100 km for the transport of 28 t materials with a CO2 emission of emission de 2.6 kg CO2 for 1 l de diesel + emission during application
			kg CO_2 ha^{-1}	5	9	13	23	
	Fate in soils	IPCC, Ref[30]	kg C kg -1	0.000	0.120	0.000	0.120	https://www.ipcc-nggip.iges.or.jp/public/2006gl/french/pdf/4_Volume4/V4_11_Ch11_N2O&CO2.pdf
		IPCC, Ref[30]	kg CO_2 ha^{-1}	0	846	0	2115	
		West *et al.* , Ref[31]		0.000	0.059	0.000	0.059	Ref[31], West, T.O., McBride, A.C. The contribution of agricultural lime to carbon dioxide emissions in the United States: dissolution, transport, and net emissions. *Agr Ecosyst Environ* . **108**, 145-154 (2005).
		West *et al.* , Ref[31]		0	415	0	1040	
GHG benefits per application at a 4 years frequency	Soil organic carbon storage		kg C ha^{-1}	3	3	3	3	Fom Paradelo et al., 2015 (Ref[36]) ; data from Versailles with a trend for an increase estimated to ΔC = +240/80 = 3 kg per application of 1t ha-1. In their synthesis, they globally observed a trend of increase of SOC in soils. "Liming does modify SOC stocks, increasing them in most cases.... Reductions in SOC have also been reported, ... Overall, these insights are deduced from published data which are still scarce, so we encourage the scientific community to synthesize unpublished SOC data from existing in situ
			kg CO_2 ha^{-1}	11	11	11	11	
	Avoided N2O emissions		kg N ha^{-1}	3.2	3.2	4.2	4.2	[illegible] 21000 Gg CO2e from 17 millions of cultivated + grassland soils that make an emission level of 1235 kg CO2e ha-1 y-1 or 3 kg N-N2O ha-1 y-1. We proposed a successive 50 %, 33 %, 25 % abatment during 3 years after application in accordance with the duration (multi-years) of the effect of liming on soil pH (Ref[34,35]) . The second calculation came from the current experiment with a mean annual emission estimated to 4 kg N-N2O ha-1 y-1. We keep the lower value coming from the first estimation.
			kg CO2e ha^{-1}	1349	1349	1749	1749	
Mean per application budget		IPCC, Ref[30]	kg CO_2 ha^{-1}	125	428	-1329	-571	
		West et al.	kg CO_2 ha^{-1}	125	859	-1329	505	
		IPCC, Ref[30]	kg C ha^{-1}	34	117	-362	-156	
		West et al.	kg C ha^{-1}	34	234	-362	138	
Mean annual budget		IPCC, Ref[30]	kg CO_2 ha^{-1}	25	86	-266	-114	
		West et al.	kg CO_2 ha^{-1}	25	172	-266	101	
		IPCC, Ref[30]	kg C ha^{-1}	7	23	-72	-31	
		West et al.	kg C ha^{-1}	7	47	-72	28	
