# Supplementary material for: Management of soil pH promotes nitrous oxide reduction and thus mitigates soil emissions of this greenhouse gas
Source: Sci Rep. 2019 Dec 27;9:20182. doi: 10.1038/s41598-019-56694-3 (PMC6934481; doi:10.1038/s41598-019-56694-3)
Supplement: Supplementary file 7 — SI_7. [file 41598_2019_56694_MOESM7_ESM.pdf]

Management of soil pH promotes nitrous oxide reduction and thus mitigates soil emissions of this greenhouse gas

Catherine Hénault[1,2 (*)], Hocine Bourennane[2], Adeline Ayzac[2], Céline Ratié[3], Nicolas Saby[3], Jean-Pierre Cohan[4], Thomas Eglin[5], Cécile Le Gall[6]

[1] Agroécologie, AgroSup Dijon, INRA, Univ. Bourgogne Franche-Comté, F-21000 Dijon, France

[2] URSOLS, INRA, 45075 Orléans, France

[3] Infosol, INRA, 45075 Orléans, France

[4]ARVALIS- Institut du Végétal Route de Châteaufort – RD 36 – ZA des Graviers

91190 – Villiers le Bacle, France

[5] ADEME, Direction Productions et Energies Durables, Service Forêts, Alimentation et Bioéconomie, F-49000 Angers, France

[6] TERRES INOVIA, Avenue Lucien Brétignières, 78850 Thiverval Grignon, France

Supplementary Information 7. Description of work conducted at the experimental sites of La Jaillière and of Presly la Noue

The E1 experiment was set up at the experimental station of La Jaillière (Arvalis, France https://www.youtube.com/watch?v=hUkYsZ4BVgk) in September 2013.

In preparation for corn silage planted in April 2014, a catch crop (vetch-oat mixture) was planted immediately after the addition of lime, up to mid-March 2014. The cover, which was well developed, was destroyed mechanically with two passes of a toothed tool. The corn was sown on 18/04/2014 (Agro Vitalo variety) using a rotary harrow and seeder with a spacing of 75 cm. Weeding was done chemically (three passes). Nitrogen fertiliser was spread once at the 3-leaf stage, with 60 kg of N ha^{-1} (33.5% ammonium nitrate). Water was added twice: 25 mm when the panicles appeared (16/07/2014) and during the blossoming of the female flowers (01/08/2014). Harvesting took place on 16/9/2014. Soft winter wheat was sown on 23/10/2014. Three inputs of nitrogen were spread in spring using ammonium nitrate at 33.5%: 70 kg of N ha^{-1} on 09/03/2015, then 50 kg of N ha^{-1} on 30/03/2015, and lastly 60 kg of N ha^{-1} on 10/05/2015. The harvest took place on 16/07/2015.

The E2 experiment was set up on 20 August 2013 and planned for the 2013-2014 campaign on a farm plot at Presly la Noue (18), on a sandy-loam soil with acid pH (5.5). The lime and manure were spread one week before seeding the crop (winter rapeseed) so as not to risk damaging the seed and jeopardising emergence. The pig manure was spread with a sprayer and was buried by a rotary tiller within two days, at a rate of 25 m^3 ha^{-1}, and the lime was applied at a rate of 1420 UVN ha^{-1}. The abundant rainfall in September and considerable damage by game jeopardised the rapeseed crop. The “control” and “lime” plot modalities were those most affected, which also led to heterogeneous stands between the modalities, possibly biasing the results of later measurements. Therefore, the decision was made to destroy the rapeseed (chemically) and plant

a spring crop. The crop chosen was brown mustard, notably because its high glucosinolate content is poorly appreciated by wild game.

The mustard was sown at the beginning of March 2014 and was covered with winter row covers to accelerate emergence and reduce the risk of insect attacks (flea beetle). However, the N_2O measurement chambers were not covered in order to avoid introducing a bias in the measures. The mustard developed well. Mineral fertilisers were spread during two events: the first input was 50 UN ha^{-1} on 2 May and the second was 50 UN ha^{-1} on 9 May. Only the "pig manure" modality plots did not receive the second input; due to the input of manure in autumn, the plots of this modality had already received an input of nitrogen exploitable by the crop. Thus, spreading the second input would have led to over-fertilisation. The first phase of monitoring was started between the end of August and the end of September 2013. Four samples were taken in order to evaluate the short-term effects of the inputs of lime and pig manure.
